# DNA barcoding of Dutch birds

**DOI:** 10.3897/zookeys.365.6287

**Published:** 2013-12-30

**Authors:** Mansour Aliabadian, Kevin K. Beentjes, C.S. (Kees) Roselaar, Hans van Brandwijk, Vincent Nijman, Ronald Vonk

**Affiliations:** 1 Department of Biology, Ferdowsi University of Mashhad, Mashhad, Iran; 2 Naturalis Biodiversity Center, Leiden, the Netherlands; 3 Department of Social Sciences, Oxford Brookes University, Oxford, UK; 4 Institute for Biodiversity and Ecosystem Dynamics, University of Amsterdam, Amsterdam, the Netherlands

**Keywords:** Aves, conservation, cytochrome *c* oxidase subunit I, COI, taxonomy

## Abstract

The mitochondrial cytochrome *c* oxidase subunit I (COI) can serve as a fast and accurate marker for the identification of animal species, and has been applied in a number of studies on birds. We here sequenced the COI gene for 387 individuals of 147 species of birds from the Netherlands, with 83 species being represented by > 2 sequences. The Netherlands occupies a small geographic area and 95% of all samples were collected within a 50 km radius from one another. The intraspecific divergences averaged 0.29% among this assemblage, but most values were lower; the interspecific divergences averaged 9.54%. In all, 95% of species were represented by a unique barcode, with 6 species of gulls and skua (*Larus* and *Stercorarius*) having at least one shared barcode. This is best explained by these species representing recent radiations with ongoing hybridization. In contrast, one species, the Lesser Whitethroat *Sylvia curruca* showed deep divergences, averaging 5.76% and up to 8.68% between individuals. These possibly represent two distinct taxa, *S. curruca* and *S. blythi*, both clearly separated in a haplotype network analysis. Our study adds to a growing body of DNA barcodes that have become available for birds, and shows that a DNA barcoding approach enables to identify known Dutch bird species with a very high resolution. In addition some species were flagged up for further detailed taxonomic investigation, illustrating that even in ornithologically well-known areas such as the Netherlands, more is to be learned about the birds that are present.

## Introduction

DNA barcoding is used as an effective tool for both the identification of known species and the discovery of new ones (Hebert et al. 2003, 2010, Savolainen et al. 2005). The core idea of DNA barcoding is based on the fact that just a small portion of a single gene, comprising a 650 to 700 bp fragment from the first half of the mitochondrial cytochrome *c* oxidase subunit I gene (COI), shows a lower intraspecific than interspecific variation. An attribute which characterizes a threshold of variation for each taxonomic group, above which a group of individuals does not belong to the same species but instead forms an intraspecific taxon. In other words, the recognition of patterns in sequence diversity of a small fragment from the mtDNA genome has led to an alternative approach for species identification across phyla.

Initially, DNA barcodes were proposed for the Animal Kingdom in 2003, when Hebert and colleagues tested a single gene barcode to identify species and coined the term ‘DNA barcoding’ (Hebert et al. 2003). Since that time COI sequences have been used as identifiers in the majority of animal phyla including vertebrates (e.g. Hebert et al. 2004, Ward et al. 2005, Kerr et al. 2007, Smith et al. 2008, Nijman and Aliabadian 2010, Luo et al. 2011) and invertebrates (Hajibabaei et al. 2006, Bucklin et al. 2011, Hausmann et al. 2011). In recent years, the practical utility of DNA barcodes proved to be an appealing tool to help resolve taxonomic ambiguity (Hebert et al. 2004, 2010), to screen biodiversity (e.g. Plaisance et al. 2009, Naro-Maciel et al. 2009, Grant et al. 2011), and to support applications in conservation biology (Neigel et al. 2007, Rubinoff 2006, Dalton and Kotze 2011).

Birds are among the best-known classes of animals and thus provide a taxonomically good model for analyzing the applicability of DNA barcoding. In the last seven years some 30 scientific papers have been published on the DNA barcoding of bird species, which combined have been cited 500 times (V. Nijman, unpubl. data April 2013). Most of the studies have shown that from this small fragment of DNA, individuals have been identified down to species level for 94% of the species in Scandinavian birds (Johnsen et al. 2010), 96% in Nearctic birds (Kerr et al. 2009a), 98% in Holarctic birds (Aliabadian et al. 2009) and 99% in Argentinean and South Korean birds (Kerr et al. 2009a, Yoo et al. 2006). Species delineation relying on the use of theshold set to differentiate between intraspecific variation and interspecific divergence has been criticized as leading to too unacceptable high error rates especially in incompletely samples groups (Meyer and Paulay 2005). However, even the critics of DNA barcoding concede that DNA barcoding holds promise for identification in taxonomically well-understood and thoroughly sampled clades. Birds are taxonomically well-known, especially those of the Western Palearctic to which the Netherlands, our study area, forms part. As noted by Taylor and Harris (2012), compared to other taxa that have been subjected to DNA barcoding, DNA barcoding studies of birds tend to represent aggregations of very large number of bird species barcodes. These often include (near) cosmopolitan species with samples from distant geographic locations potentially increasing the amount of interspecific variation in COI.

Here we explore the efficiency of identifying species using DNA barcoding from a large set of sympatric bird species in the Netherlands. Compared to previous studies on birds, our study area covers a very small geographic area, allowing to directly test the functionality of DNA barcoding ‘in one’s backyard’.

## Methods

### Sampling

The Netherlands is a small, densely populated country in northwestern Europe, with a land surface area of some 34 000 km^2^, and ornithologically it is arguable one of the best-covered countries (Sovon 2002). The tissue samples used for sequencing were collected from breeding areas in the Netherlands, excluding oversees dependencies. Given the small size of the country some 95% of the samples were collected within a 50 km-radius of each other. Samples were part of the tissue collection of the Zoological Museum of Amsterdam (ZMA), which were recently relocated and deposited in the Naturalis Biodiversity Center, Leiden. Most were collected in the period 2000–2012 by a network of volunteers, ringers, airport staff, and bird asylums; no birds were specifically collected or killed to be included in the collection of the ZMA. Species and subspecies identification was based on morphology and when necessary, external measurements. These identifications were done by authors HvB and CSR, with the help of Tineke G. Prins. Individual birds were frozen upon arrival to be thawed and skinned at a later date, and indeed many birds arrived frozen. Samples were mostly taken from the bird’s pectorial muscles, because of its size and easy access, and stored in 96% ethanol. Species nomenclature follows the taxonomy of Dickinson (2003). The complete list of sampled specimens including information about vouchers and trace files is available from the project ‘Aves of the Netherlands’ at the BOLD website (http://www.barcodinglife.com/).

### PCR and sequencing

The tissue samples were subsampled and subjected to DNA extraction using DNeasy Blood & Tissue Kit (Qiagen) following the manufacturer’s protocol. PCR and sequencing reactions were performed, mainly following the same protocols described in Förschler et al. (2010), but with some minor modifications. Polymerase chain reaction (PCR) amplifications were initially performed using standard primers BirdF1 (TTCTCCAACCACAAAGACATTGGCAC) and BirdR1 (ACGTGGGAGATAATTCCAAATCCTG). When amplification was unsuccessful, alternate reverse primer BirdR2 (ACTACATGTGAGATGATTCCGAATCCAG) was used in combination with BirdF1 or alternate primer pair CO1-ExtF (ACGCTTTAACACTCAGCCATCTTACC) and CO1-ExtR (AACCAGCATATGAGGGTTCGATTCCT) was used (Hebert et al. 2004, Johnsen et al. 2010). All PCRs were run under the following thermal cycle program: 3 min at 94 °C followed by 40 cycles of 15 s at 94 °C, 30 s at 50 °C and 40 s at 72 °C, and a final elongation of 5 min at 72 °C. For each reaction the PCR mixture consisted of 2.5 µl Qiagen Coral Load 10 × PCR buffer, 1.0 µl of each 10 mM primer, 0.5 µl 2.5 mM dNTPs, 0.25 µl 5 U/µl QiagenTaq DNA polymerase, 18.75 µl milliQ and 1.0 µl template DNA for a total volume of 25 µl. Bi-directional sequencing was performed for all specimens at Macrogen. We checked the possible amplification of pseudogenes (Numts) by translating the protein coding genes into amino acids sequences, but we did not observe any unexpected stop codons, frameshifts or unusual amino acidic substitutions. Furthermore we amplified a longer sequence of the COI gene with primers (CO1-ExtF and CO1-ExtR) for selected samples, and also here we did not see any indication of pseudogene co-amplification. Lijtmaer et al. (2012) found that, in birds, full-length COI pseudogenes are uncommon noting that they might be more frequently encountered when working with avian blood samples as opposed to muscle tissue samples (as used in here).

### Data analysis

Sequences shorter than 500 bp and containing more than 10 ambiguous nucleotides were excluded from the analyses. All sequences have been deposited in GenBank (Accession numbers KF946551 to KF946937). A full list of the museum vouchers, for all specimens applied in this study, is provided in Appendix – Table 1.

For all sequence comparisons, the Kimura 2-parameter (K2P) model was used, because it is shown to be the best metric to compare closely related taxa (Nei and Kumar 2000, but for a contrasting view see Srivathsan and Meier 2012). Average intraspecific distances were calculated for those species that were represented by at least two specimens using MEGA5 software (Tamura et al. 2011).

For a group of birds that expressed a larger than expected intraspecific variation, the *Sylvia* warblers, we created a phylogenetic tree and created a haplotype network. We chose GTR+G+I as the best-fitting model of nucleotide substitution based on its Akaike’s information criterion as implemented in JModelTest v0.1.1 (Posada 2008). A maximum likelihood (ML) tree was constructed in PAUP* v4.0b10 (Swofford 2002) using a heuristic search with the tree-bisection-reconnection branch-swapping algorithm and random addition of taxa. Relative branch support was evaluated with 500 bootstrap replicates (Felsenstein 1985). A minimum spanning haplotype network was constructed using a statistical parsimony network construction approach as implemented in TCS software package (Clement et al. 2000). This programme calculates the number of mutational steps by which pairwise haplotypes differ and computes the probability of parsimony (Templeton et al. 1992) for pairwise differences until the probability exceeds 0.95. The number of mutational differences associated with the probability just before the 0.95 cut-off point is then the maximum number of mutational connections between pairs of sequences justified by the parsimony criterion; these justified connections are applied in the haplotype network (Clement et al. 2000).

### Results

**Figure 1.**
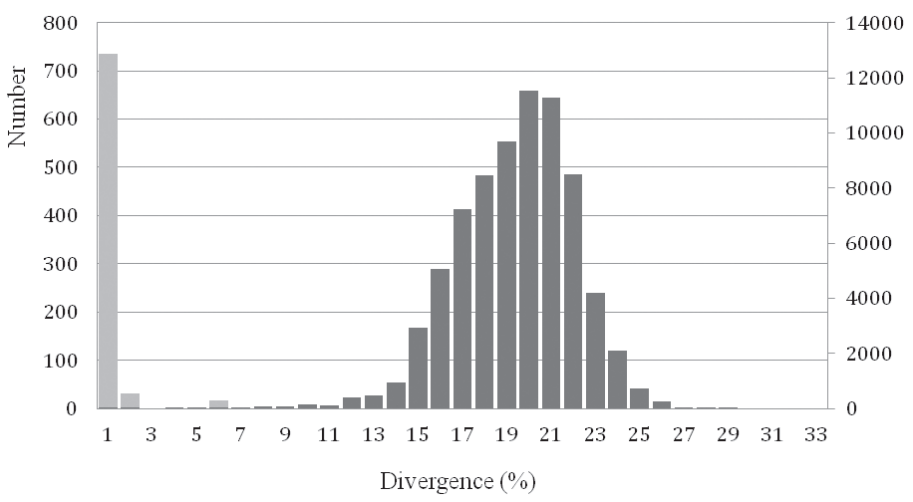
Comparisons of K2P-pairwise distances based on the COI gene of 141 species of birds from the Netherlands, showing a clear barcoding gap. Interspecific distances are indicated with light grey bars and intraspecific distances with dark grey bars. Left Y-axis: numbers of intraspecific comparisons; Right Y-axis: numbers of interspecific comparisons.

A total of 387 sequences for 141 species (representing at least 158 taxa) were retrieved, including 52% of the breeding bird species in the Netherlands (Supplementary table 1). The average number of sequences per species was 3.36 (range 1-13), with 83 species (59%) represented by more than two sequences. The mean K2P-divergence within species bears no significant relationship with sample sizes, i.e. number of sequences per species (R^2^ = 0.001, p = 0.465). The mean intraspecific K2P-distance was 0.29% (range 0-8.68%) some 30 times lower than the mean intrageneric K2P-distances (9.54%, range 0-27.71%) (Table 1, Figure 1).

**Table 1.** Comparisons of K2P-pairwise distances within various taxonomic levels for 83 species of birds from the Netherlands for which two or more sequences were available. Distances are expressed in percentages.

	Individuals	Taxa	Comparisons	Distances		
				Minimum	Mean ± S.E.M.	Maximum
Within Species	340	83	805	0	0.294±0.001	8.683
Within Genera	203	23	794	0	9.544±0.004	15.849
Within Families	282	20	2519	5.809	14.467±0.001	20.473

In general, 95% of species (134 species) showed a unique DNA barcode (these included the 58 species for which we only sequenced single individuals), while six congeneric species shared the same barcode and the mean intraspecific distance of them fell well below the threshold of species based on distance-based criterion (Hebert et al. (2003) 10 × rule). These congeneric species mostly included circumpolar species with close morphological similarities (Table 2).

**Table 2.** Bird species (Charadriiformes) from the Netherlands with one or more shared DNA barcodes (K2P-distances of 0%). For a detailed breakdown of the individual samples involved see Appendix – Table 2.

Family	Species	Nearest species	Mean K2P-distance (%)
Laridae	Herring Gull *Larus argentatus*	Yellow-legged Gull *Larus michahellis*	0.14
	Lesser Black-backed Gull *Larus fuscus*	Caspian Gull *Larus cachinnans*	0
	Iceland Gull *Larus glaucoides*	Caspian Gull *Larus cachinnans*	0
	Glaucous Gull *Larus hyperboreus*	Yellow-legged Gull *Larus michahellis*	0.58
	Yellow-legged Gull *Larus michahellis*	Caspian Gull *Larus cachinnans*	0
Stercorariidae	Great Skua *Stercorarius skua*	Pomarine Skua *Stercorarius pomarinus*	0.30

**Figure 2.**
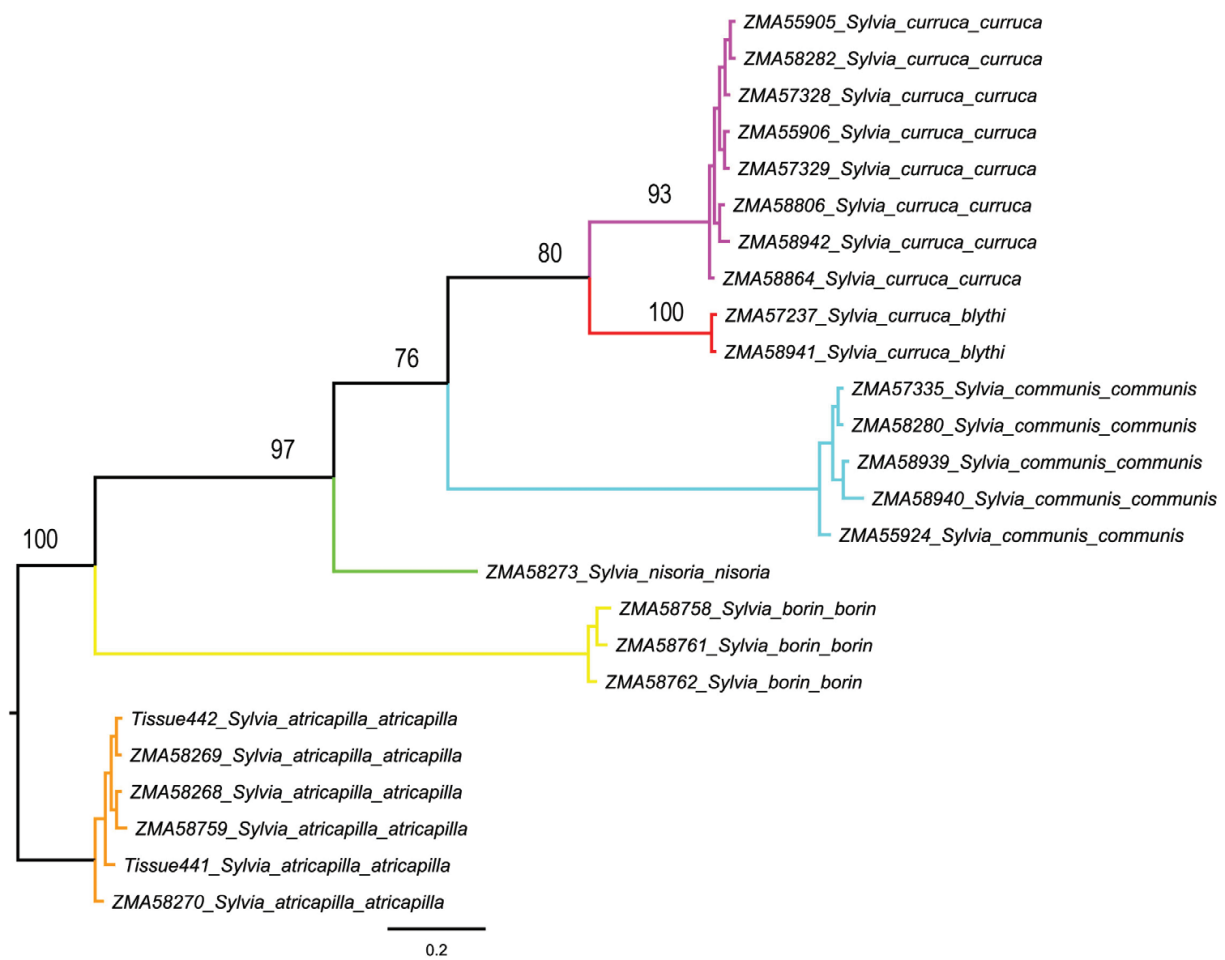
Phylogenetic relationships of two putative subspecies of Lesser Whitethroat, i.e. the Western Lesser Whitethroat *Sylvia curruca curruca* and the Northeastern Lesser Whitethroat *Sylvia curruca blythi* from the Netherlands, based on analysis of 694 bp of the mitochondrial cytochrome *c* oxidase subunit I gene (COI). Bootstrap values are given for the maximum likelihood (ML) analysis.

**Figure 3.**
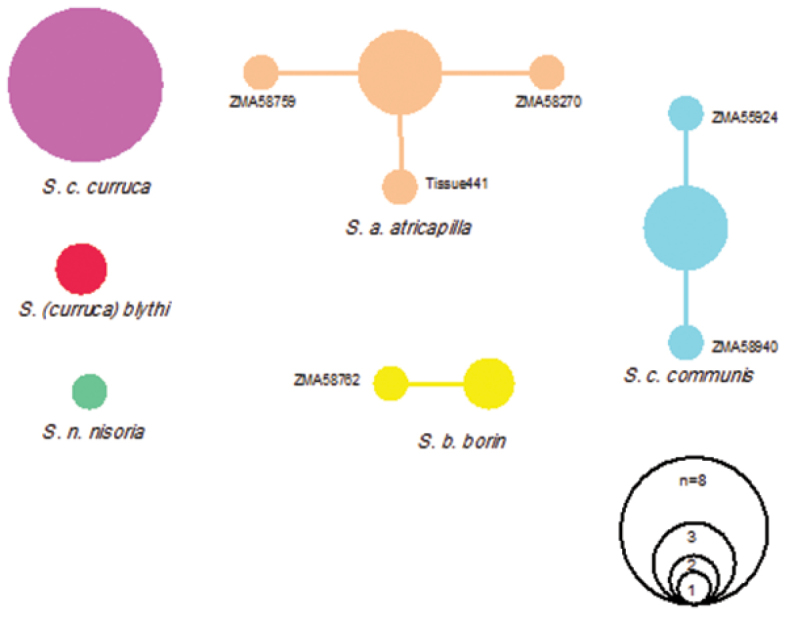
Haplotype networks constructed with statistical parsimony based on 694 bp of the mitochondrial cytochrome *c* oxidase subunit I gene (COI) of the *Sylvia* group (25 individuals). Each circle represents one haplotype; size of circles is proportional to haplotype frequency.

Although most species possessed low intraspecific distances, one species showed high intraspecific K2P-distances clearly above the threshold of 2 to 3 per cent sequence divergence in our data set. This is the Lesser Whitethroat *Sylvia curruca*, with a mean interspecific divergence of 5.76% and a maximum interspecific distance of 8.68%. Two subspecies occur in the Netherlands, i.e. the Western Lesser Whitethroat *Sylvia curruca curruca* and, as a migrant, the Northeastern Lesser Whitethroat *Sylvia curruca blythi*. Both are morphologically somewhat distinct, with compared to the nominate *Sylvia curruca blythi* having a paler top of the head, separated from face by a white supercilium, and geographically the nominate occupies the western part of the species range and *Sylvia curruca blythi* the eastern part. A maximum likelihood tree for these two taxa based on K2P-model is presented in Figure 2. Two different haplotype networks, one each for *Sylvia curruca curruca* and *Sylvia curruca blythi* were recovered by TCS (Figure 3), and given the large genetic distances between their haplotypes, the two taxa are not included in the same haplotype network.

## Discussion

We here present the results of a modest effort to barcode the avifauna of the Netherlands. In terms of DNA barcoding of birds, the Netherlands form the southernmost part of one of the most densely sampled regions globally (Lijtmaer et al. 2012: figure 1). In addition, many of the species that overwinter in the country originate equally well-sampled regions to the north. As such our study adds to a growing number of studies allowing us to build up comprehensive public libraries of bird barcodes. Combined these allow us to explore new lines of scientific inquiry and practical applications (Hebert et al. 2010, Lijtmaer et al. 2012, but see Ebach and Carvalho 2010). The collection of our samples was done as part of the museum’s standard collection management of newly obtained material, and as such sample collection was inexpensive and required little effort in terms of manpower. All birds were collected and processed in the Netherlands and did not require specific permits other than the ones already required to curate the collections.

Recently, Taylor and Harris (2012) expressed the opinion that proponents of DNA barcoding consistently fail to recognize its limitations (including, but not restricted to, the functioning of COI as a universal barcoding gene, whether its use is to be restricted to species identification only or whether it has a role in species discovery and delimitation and the failure to have sufficient systems in place to deal with the large amounts of data generated), do not evolve their methodologies, and do not embrace the possibilities that next-generation sequencing offers. We agree that DNA barcoding will not offer a panacea for all the issues Taylor and Harris (2012) raised, or indeed some of its earlier critics (Will et al. 2005, Moritz and Cicero 2004) but we point out that for this was probably never the intention of DNA barcoding when envisaged some ten years ago. Irrespective of the aims and goals of DNA barcoding as a ‘global enterprise’ (Ebach and Carvalho 2010), we found it a useful tool in our studies on birds (cf. Baker et al. 2009). The bird collection of the Zoological Museum Amsterdam, and our sample reported in this study, was well-curated by knowledgeable staff, with a very high degree of taxonomic certainty attached to each individual specimen. We see immense value to having a DNA barcoding dataset linked to this reference collection. As such this work has added to the growing library of DNA barcodes of bird species of the world and subsequent improvement in our knowledge of biodiversity.

The mean intraspecific divergences found in the birds of the Netherlands (0.29%, based on 147 species) is congruent with that of for instance Argentina (0.24%, 500 species), North America (0.23%, 643 species) and the Holarctic (0.24%, 566 species) (Kerr et al. 2009a, Aliabadian et al. 2009). More importantly, like other studies on birds, the efficiency of DNA barcode sequences to identify species is high, showing a clear barcoding gap (Figure 1), and overall it seems that for birds typically 95% or more of the species can be identified (Hebert et al. 2003, Johnsen et al. 2010, Kerr et al. 2009a, b, Yoo et al. 2006, Aliabadian et al. 2009).

Most DNA barcoding studies of birds flag a small number of deep divergences (e.g. Johnsen et al. 2010, Kerr et al. 2009b, Aliabadian et al. 2009, Nijman and Aliabadian 2013), in our study involving the two subspecies of *Sylvia curruca*, where the two lineages diverge almost 6%. Similar results were found by Olsson et al. (2013) when analyzing the cytochrome *b* gene for these two taxa, with distances in the order of 11-14%. Based on COI sequences, the two taxa appear to be sister taxa, albeit with a relatively low support (Figure 3), but no other members of the *Sylvia curruca* were included in the analysis. In contrast, having included a range of other members of this complex, Olsson et al. (2013) found *curruca* and *blythi* not to be sister taxa. Olsson et al. (2013: 81) concluded that while “due to their morphological similarity it is unclear where their ranges meet, [o]ur data suggest that *blythi* is a valid taxon, not closely related to *curruca*. It has its closest relatives to the south-east [Asia], and may have colonised the eastern taiga from this direction, ultimately coming into contact with *curruca*”. When it comes to drawing conclusion from their work with respect to taxomomy, Olsson et al. (2013) were, in our view correctly, cautious. They noted that the *Sylvia curruca* complex comprised up to 13 taxa with little consensus as to circumscription and taxonomic rank. Of these, morphologically some taxa are very similar, including *Sylvia curruca curruca* and *Sylvia curruca blythi*, and the apparent conflict between morphology and phylogeny (based in their case on cyt *b* and in our study on COI) can be explained in different ways. One would be to accept the single mitochondrial gene trees at face value in which case the morphological similarities in pelage coloration may be a result of parallel evolution possibly in response to adaptations to similar temperate forest habitats – both taxa are then best treated as different species. Alternatively, the mitochondrial gene trees do not reflect the species tree and, based on morphological similarities, *Sylvia curruca curruca* and *Sylvia curruca blythi* are best treated as sister taxa (either as one or two species). Their divergent position on the mitochondrial gene tree, and the large genetic distances between these taxa, are due to ancient mitochondrial introgression. In either case, working with single mitochondrial markers cannot not resolve this issue and a more integrative approach ideally involving the analysis of nuclear genes is paramount.

Those cases where we found species sharing the same DNA barcodes were small in number but not insignificant. Seven of the eight cases involved closely related gulls with partially overlapping ranges, or allopatric distributions, that are part of a recent Holarctic radiation (Liebers-Helbig et al. 2010). Alternatively, the sharing of DNA barcodes may be due to hybridization or, perhaps less likely, misidentification. Likewise, skuas are part of a recent radiation with, just like gulls, frequent hybridization between species (Ritz 2009). DNA barcoding using a relative slowly evolving maternally inherited gene, with, compared to other mitochondrial genes, small amounts of rate heterogeneity (Pacheco et al. 2011), will, on its own, not be able to differentiate between these taxa.

We conclude that DNA barcoding approach makes it possible to identify known Dutch bird species with a very high resolution. Although some species were flagged for further detailed taxonomic investigation, our study reaffirms once more that a short segment of COI gene can be used to handle large number of taxa and aid in detecting overlooked taxa and hybridizing species with low deep barcode divergences.

## Appendix

**Supplementary table 1.** List of all Dutch birds that have been sequenced in this study, with voucher numbers and collection localities. Note that specimens from which only tissue samples have been taken have not been given a collection number, sine loco refers to specimens collected in the Netherlands but without a precise named collection locality. Localities in the province of Friesland are listed with their Dutch name first, followed by their Frisian name. Coordinates are given in decimal degrees.

Species or subspecies	ZMA number	Preparation	Locality	Coordinates	Access numbers
*Accipiter gentilis gentilis*	ZMA58297	skin	Zaandam	52.25N, 4.49E	KF946551
*Accipiter gentilis gentilis*	ZMA58724	skin	De Rips	51.32N, 5.48E	KF946552
*Accipiter nisus nisus*	ZMA58243	skin	Malden	51.47N, 5.52E	KF946553
*Accipiter nisus nisus*	ZMA58245	skin	Helden	51.21N, 5.55E	KF946554
*Accipiter nisus nisus*	ZMA58246	skin	Reuver	51.17N, 6.04E	KF946555
*Accipiter nisus nisus*	ZMA58247	skin	Culemborg	51.55N, 5.15E	KF946556
*Accipiter nisus nisus*	ZMA58248	skin	Amsterdam	52.21N, 4.53E	KF946557
*Accipiter nisus nisus*	ZMA58741	skin	Amsterdam	52.21N, 4.53E	KF946558
*Accipiter nisus nisus*	ZMA58742	skin	Montfort	51.07N, 5.56E	KF946559
*Accipiter nisus nisus*	ZMA58743	skin	Belfeld	51.18N, 6.08E	KF946560
*Accipiter nisus nisus*	ZMA58744	skin	Laren	52.11N, 6.22E	KF946561
*Accipiter nisus nisus*	ZMA58745	skin	Almere	52.22N, 5.13E	KF946562
*Accipiter nisus nisus*	ZMA58746	skin	Venlo	51.21N, 6.11E	KF946563
*Acrocephalus palustris*	ZMA56679	skin	Harderbroek reserve	52.22N, 5.35E	KF946564
*Acrocephalus palustris*	ZMA58811	skin	Castricum	52.32N, 4.36E	KF946565
*Acrocephalus schoenobaenus*	ZMA58278	skin	Almere	52.22N, 5.13E	KF946566
*Acrocephalus schoenobaenus*	ZMA58809	skin	Almere	52.22N, 5.13E	KF946567
*Acrocephalus schoenobaenus*	ZMA58810	skin	Castricum	52.32N, 4.36E	KF946568
*Acrocephalus schoenobaenus*	ZMA58862	skin	Wassenaar	53.08N, 5.53E	KF946569
*Acrocephalus scirpaceus scirpaceus*	ZMA58277	skin	Oostvaardersdijk	52.29N, 5.23E	KF946570
*Acrocephalus scirpaceus scirpaceus*	ZMA58725	skin	Schermerhorn	52.36N, 4.54E	KF946571
*Acrocephalus scirpaceus scirpaceus*	ZMA58727	skin	Lelystad	52.29N, 5.24E	KF946572
*Acrocephalus scirpaceus scirpaceus*	ZMA58728	skin	Lelystad	52.29N, 5.24E	KF946573
*Acrocephalus scirpaceus scirpaceus*	ZMA58729	skin	Castricum	52.32N, 4.36E	KF946574
*Acrocephalus scirpaceus scirpaceus*	ZMA58863	skin	Lauwersmeer	53.22N, 6.14E	KF946575
*Acrocephalus scirpaceus scirpaceus*	ZMA58937	skin	Lelystad	52.29N, 5.24E	KF946576
*Acrocephalus scirpaceus scirpaceus*	ZMA58938	skin	Purmerend	52.28N, 4.58E	KF946577
*Aegithalos caudatus europaeus*	ZMA57353	skin	Westenschouwen	51.41N, 3.42E	KF946578
*Aegithalos caudatus europaeus*	ZMA57354	skin	Westenschouwen	51.41N, 3.42E	KF946579
*Aegithalos caudatus europaeus*	ZMA57356	skin	Hilversum	52.13N, 5.09E	KF946580
*Aegithalos caudatus europaeus*	ZMA58804	skin	Castricum	52.32N, 4.36E	KF946581
*Alcedo atthis ispida*	ZMA56216	skin	Haelen	51.13N, 5.56E	KF946582
*Alcedo atthis ispida*	ZMA57341	skin	Purmerland	52.28N, 4.55E	KF946583
*Alcedo atthis ispida*	ZMA57342	skin	Alkmaar	52.38N, 4.44E	KF946584
*Alcedo atthis ispida*	ZMA57343	skin	Utrecht	52.03N, 5.08E	KF946585
*Alcedo atthis ispida*	ZMA58869	skin	Leeuwarden/Ljouwert	53.13N, 5.45E	KF946586
*Alle alle alle*	ZMA58842	skin	Amsterdam	52.21N, 4.53E	KF946587
*Alle alle alle*	ZMA58917	skin	Amsterdam	52.21N, 4.53E	KF946588
*Alle alle alle*	ZMA58918	skin	Den Helder	52.55N, 4.46E	KF946589
*Anas acuta*	ZMA58228	skin	Vlieland Island	53.15N, 4.59E	KF946590
*Anas strepera strepera*	ZMA58913	skin	Driebond Polder	53.11N, 6.37E	KF946591
*Anthus spinoletta spinoletta*	ZMA58279	skin	Lelystad	52.29N, 5.24E	KF946592
*Anthus spinoletta spinoletta*	ZMA64552	skin	Castricum	52.32N, 4.36E	KF946593
*Anthus trivialis trivialis*	Tissue553	DNA sample	Castricum	52.32N, 4.36E	KF946594
*Apus apus apus*	ZMA58717	skin	Tegelen	51.19N, 6.09E	KF946595
*Ardea cinerea cinerea*	Tissue434	DNA sample	Leeuwarden/Ljouwert	53.13N, 5.45E	KF946596
*Ardea cinerea cinerea*	Tissue435	DNA sample	Leeuwarden/Ljouwert	53.13N, 5.45E	KF946597
*Asio flammeus flammeus*	ZMA58253	skin	Texel Island	53.04N, 4.43E	KF946598
*Asio otus otus*	Tissue455	DNA sample	Leeuwarden/Ljouwert	53.13N, 5.45E	KF946599
*Asio otus otus*	ZMA58233	skin	Purmerend	52.28N, 4.58E	KF946600
*Asio otus otus*	ZMA58234	skin	Zutphen	52.07N, 6.12E	KF946601
*Athene noctua vidalii*	ZMA58493	skin	Heerhugowaard	52.4N, 4.51E	KF946602
*Athene noctua vidalii*	ZMA58294	skin	Blerick	51.21N, 6.08E	KF946603
*Bombycilla garrulus garrulus*	ZMA56300	skin	Amsterdam	52.21N, 4.53E	KF946604
*Bombycilla garrulus garrulus*	ZMA56301	wings	Texel Island	53.04N, 4.43E	KF946605
*Bombycilla garrulus garrulus*	ZMA58301	wings	Hellendoorn	52.23N, 6.26E	KF946606
*Bombycilla japonica*	ZMA58302	skin	Amsterdam	52.21N, 4.53E	KF946607
*Buteo buteo buteo*	Tissue461	DNA sample	Leeuwarden/Ljouwert	53.13N, 5.45E	KF946608
*Buteo buteo buteo*	ZMA58238	skin	Wieringermeer	52.54N, 5.01E	KF946609
*Buteo buteo buteo*	ZMA58239	skin	De Rips	51.32N, 5.48E	KF946610
*Buteo buteo buteo*	ZMA58781	wing	Leeuwarden/Ljouwert	53.13N, 5.45E	KF946611
*Buteo buteo buteo*	ZMA58828	skin	Wartena	52.12N, 4.3E	KF946612
*Buteo buteo buteo*	ZMA58920	wings	Rolde	52.58N, 6.38E	KF946613
*Calidris alpina alpina*	ZMA58700	skin	Schiermonnikoog Island	53.29N, 6.11E	KF946614
*Calonectris diomedea borealis*	ZMA57255	skin	Lith	51.47N, 5.26E	KF946615
*Carduelis cannabina cannabina*	ZMA58911	skin	Noordijk	52.08N, 6.34E	KF946616
*Carduelis carduelis*	ZMA58866	skin	Schiermonnikoog Island	53.29N, 6.11E	KF946617
*Carduelis chloris chloris*	ZMA57337	skin	Cadier en Keer	50.49N, 5.46E	KF946618
*Carduelis chloris chloris*	ZMA58947	skin	Goor	52.14N, 6.34E	KF946619
*Carduelis flammea cabaret*	ZMA57248	skin	Kennemerduinen	52.42N, 4.58E	KF946620
*Carduelis flammea cabaret*	ZMA58283	skin	Westenschouwen	51.41N, 3.42E	KF946621
*Carduelis flammea flammea*	ZMA57251	skin	Kennemerduinen	52.42N, 4.58E	KF946622
*Carduelis flammea flammea*	ZMA64564	skin	Castricum	52.32N, 4.36E	KF946623
*Carduelis flavirostris*	ZMA57253	skin	Castricum	52.32N, 4.36E	KF946624
*Carduelis flavirostris*	ZMA57254	skin	Castricum	52.32N, 4.36E	KF946625
*Carduelis spinus*	ZMA55904	skin	Nijverdal	52.22N, 6.28E	KF946626
*Carduelis spinus*	ZMA57256	skin	Westenschouwen	51.41N, 3.42E	KF946627
*Carduelis spinus*	ZMA58286	skin	Hellendoorn	52.23N, 6.26E	KF946628
*Certhia brachydactyla megarhyncha*	ZMA57322	skin	Hellendoorn	52.23N, 6.26E	KF946629
*Certhia brachydactyla megarhyncha*	ZMA57323	skin	Lekkerkerk	51.53N, 4.41E	KF946630
*Certhia brachydactyla megarhyncha*	ZMA57325	skin	Wageningen	51.58N, 5.38E	KF946631
*Certhia brachydactyla megarhyncha*	ZMA57326	skin	Zeist	52.05N, 5.16E	KF946632
*Certhia brachydactyla megarhyncha*	ZMA57327	skin	Heiloo	52.36N, 4.44E	KF946633
*Certhia brachydactyla megarhyncha*	ZMA58805	skin	Castricum	52.32N, 4.36E	KF946634
*Certhia brachydactyla megarhyncha*	ZMA58949	skin	Lekkerkerk	51.53N, 4.41E	KF946635
*Certhia brachydactyla megarhyncha*	ZMA64563	skin	Castricum	52.32N, 4.36E	KF946636
*Charadrius hiaticula*	Tissue452	DNA sample	Leeuwarden/Ljouwert	53.13N, 5.45E	KF946637
*Circus aeruginosus aeruginosus*	ZMA58780	skin	Leeuwarden/Ljouwert	53.13N, 5.45E	KF946638
*Circus aeruginosus aeruginosus*	ZMA58826	skin	Eibergen	52.06N, 6.37E	KF946639
*Circus aeruginosus aeruginosus*	ZMA58874	wings	Zuid-Flevoland	52.26N, 5.16E	KF946640
*Coccothraustes coccothraustes*	ZMA56212	skin	Laag Keppel	51.59N, 6.13E	KF946641
*Corvus corax corax*	ZMA57144	skin	Appelscha/Appelskea	52.55N, 5.2E	KF946642
*Coturnix coturnix coturnix*	ZMA58775	skin	Deventer	52.15N, 6.11E	KF946643
*Coturnix coturnix coturnix*	ZMA58776	skin	Het Bildt	53.17N, 5.4E	KF946644
*Cuculus canorus canorus*	ZMA56681	skin	Bergen	52.4N, 4.41E	KF946645
*Cuculus canorus canorus*	ZMA64549	skin	Alkmaar	52.38N, 4.44E	KF946646
*Delichon urbicum*	ZMA56215	skin	Sea		KF946647
*Delichon urbicum urbicum*	ZMA55919	skin	Nieuwegein	52.01N, 5.05E	KF946648
*Delichon urbicum urbicum*	ZMA58300	wings	Lage Zwaluwe	51.42N, 4.42E	KF946649
*Delichon urbicum urbicum*	ZMA58870	skin	Leeuwarden/Ljouwert	53.13N, 5.45E	KF946650
*Dendrocopos major pinetorum*	ZMA58803	skin	Oudkerk/Aldtsjerk	53.15N, 5.53E	KF946651
*Dryocopus martius martius*	ZMA58766	skin	Tegelen	51.19N, 6.09E	KF946652
*Emberiza citrinella citrinella*	ZMA57257	skin	Westenschouwen	51.41N, 3.42E	KF946653
*Emberiza melanocephala*	ZMA56996	skin	Bovenkerk	52.17N, 4.49E	KF946654
*Emberiza pusilla*	ZMA58859	skin	Schiermonnikoog Island	53.29N, 6.11E	KF946655
*Emberiza pusilla*	ZMA58860	skin	Vlieland Island	53.15N, 4.59E	KF946656
*Emberiza schoeniclus schoeniclus*	ZMA58857	skin	Noordpolderzijl	53.25N, 6.34E	KF946657
*Emberiza schoeniclus schoeniclus*	ZMA58858	skin	Oostvaardersdijk	52.29N, 5.23E	KF946658
*Erithacus rubecula rubecula*	Tissue436	DNA sample	Castricum	52.32N, 4.36E	KF946659
*Erithacus rubecula rubecula*	Tissue437	DNA sample	Castricum	52.32N, 4.36E	KF946660
*Erithacus rubecula rubecula*	ZMA58274	skin	Bloemendaal	52.24N, 4.33E	KF946661
*Erithacus rubecula rubecula*	ZMA58740	skin	Doldersum	52.52N, 6.17E	KF946662
*Falco columbarius aesalon*	ZMA58840	skin	Texel Island	53.04N, 4.43E	KF946663
*Falco columbarius aesalon*	ZMA60127	skin	Spaarndam	52.24N, 4.41E	KF946664
*Falco peregrinus peregrinus*	ZMA58872	skin	Haarlem	52.23N, 4.37E	KF946665
*Falco subbuteo subbuteo*	ZMA56231	skin	Zundert	51.28N, 4.38E	KF946666
*Falco subbuteo subbuteo*	ZMA56232	skin	Heerhugowaard	52.4N, 4.51E	KF946667
*Falco subbuteo subbuteo*	ZMA58241	skin	Hoogland	52.1N, 5.21E	KF946668
*Falco subbuteo subbuteo*	ZMA58242	skin	Texel Island	53.04N, 4.43E	KF946669
*Falco subbuteo subbuteo*	ZMA58841	skin	Amsterdam	52.21N, 4.53E	KF946670
*Falco tinnunculus tinnunculus*	Tissue456	DNA sample	Leeuwarden/Ljouwert	53.13N, 5.45E	KF946671
*Falco tinnunculus tinnunculus*	ZMA58296	skin	Zaandam	52.25N, 4.49E	KF946672
*Falco tinnunculus tinnunculus*	ZMA58752	skin	Maasbree	51.21N, 6.03E	KF946673
*Falco tinnunculus tinnunculus*	ZMA58754	skin	Boekend	51.22N, 6.06E	KF946674
*Falco tinnunculus tinnunculus*	ZMA58774	skin	Leeuwarden/Ljouwert	53.13N, 5.45E	KF946675
*Falco tinnunculus tinnunculus*	ZMA58837	skin	Westzaan	52.26N, 4.46E	KF946676
*Falco tinnunculus tinnunculus*	ZMA58838	skin	Leeuwarden/Ljouwert	53.13N, 5.45E	KF946677
*Falco tinnunculus tinnunculus*	ZMA58839	wings	Reutum	52.23N, 6.5E	KF946678
*Falco vespertinus*	ZMA58773	skin	Leeuwarden/Ljouwert	53.13N, 5.45E	KF946679
*Ficedula hypoleuca muscipeta*	ZMA55913	skin	Otterlo	52.04N, 5.5E	KF946680
*Ficedula hypoleuca muscipeta*	ZMA57239	skin	Markelo	52.14N, 6.3E	KF946681
*Ficedula hypoleuca muscipeta*	ZMA57320	skin	Garderen	52.12N, 5.43E	KF946682
*Ficedula hypoleuca*	ZMA58865	skin	Eemshaven	53.26N, 6.52E	KF946683
*Fratercula arctica grabae*	ZMA56226	skin	Texel Island	53.04N, 4.43E	KF946684
*Fratercula arctica grabae*	ZMA58226	skin	Texel Island	53.04N, 4.43E	KF946685
*Fratercula arctica grabae*	ZMA58227	skin	Hondsbossche Zeewering	52.44N, 4.38E	KF946686
*Fringilla coelebs coelebs*	ZMA58948	skin	Goor	52.14N, 6.34E	KF946687
*Fringilla montifringilla*	Tissue449	DNA sample	Leeuwarden/Ljouwert	53.13N, 5.45E	KF946688
*Fulmarus glacialis auduboni*	ZMA56235	wings	Hondsbossche Zeewering	52.44N, 4.38E	KF946689
*Fulmarus glacialis glacialis*	ZMA60119	skin	Neeltje Jans	51.37N, 3.41E	KF946690
*Fulmarus glacialis glacialis*	ZMA60120	skin	Texel Island	53.04N, 4.43E	KF946691
*Fulmarus glacialis glacialis*	ZMA60121	skin	Hondsbossche Zeewering	52.44N, 4.38E	KF946692
*Fulmarus glacialis glacialis*	ZMA60123	skin	Ameland Island	53.27N, 5.39E	KF946693
*Fulmarus glacialis glacialis*	ZMA60124	skin	Ameland Island	53.27N, 5.39E	KF946694
*Fulmarus glacialis glacialis*	ZMA60125	skin	Hondsbossche Zeewering	52.44N, 4.38E	KF946695
*Fulmarus glacialis glacialis*	ZMA60126	skin	Petten	52.46N, 4.38E	KF946696
*Fulmarus glacialis*	ZMA58737	skin	Vlieland Island	53.15N, 4.59E	KF946697
*Gallinula chloropus chloropus*	Tissue105	DNA sample	Wijde Wormer	52.28N, 4.53E	KF946698
*Gallinula chloropus chloropus*	Tissue110	DNA sample	Wijde Wormer	52.28N, 4.53E	KF946699
*Garrulus glandarius glandarius*	ZMA58306	wings	Amsterdam	52.21N, 4.53E	KF946700
*Gavia immer*	Tissue214	DNA sample	Bergen aan Zee	52.39N, 4.37E	KF946701
*Haematopus ostralegus ostralegus*	Tissue458	DNA sample	Leeuwarden/Ljouwert	53.13N, 5.45E	KF946702
*Haematopus ostralegus ostralegus*	Tissue459	DNA sample	Leeuwarden/Ljouwert	53.13N, 5.45E	KF946703
*Hirundo rustica rustica*	Tissue450	DNA sample	Leeuwarden/Ljouwert	53.13N, 5.45E	KF946704
*Hirundo rustica rustica*	Tissue451	DNA sample	Leeuwarden/Ljouwert	53.13N, 5.45E	KF946705
*Hirundo rustica rustica*	ZMA56214	skin	Amstelveen	52.18N, 4.53E	KF946706
*Hirundo rustica rustica*	ZMA58289	skin	Appelscha/Appelskea	52.55N, 5.2E	KF946707
*Hirundo rustica rustica*	ZMA58290	skin	Appelscha/Appelskea	52.55N, 5.2E	KF946708
*Hirundo rustica rustica*	ZMA58696	skin	Rijswijk	51.57N, 5.21E	KF946709
*Hirundo rustica rustica*	ZMA58802	skin	Noordbergum/Noardburgum	53.13N, 6E	KF946710
*Jynx torquilla torquilla*	ZMA56213	skin	Aarle-Rixtel	51.3N, 5.39E	KF946711
*Jynx torquilla torquilla*	ZMA57330	skin	Limmen	52.34N, 4.41E	KF946712
*Jynx torquilla torquilla*	ZMA58303	wings	Belfeld	51.18N, 6.08E	KF946713
*Jynx torquilla torquilla*	ZMA58873	skin	Wilnis	52.11N, 4.54E	KF946714
*Larus argentatus argenteus*	ZMA58921	wings	Eemshaven	53.26N, 6.52E	KF946715
*Larus argentatus*	Tissue433	DNA sample	Leeuwarden/Ljouwert	53.13N, 5.45E	KF946716
*Larus cachinnans*	ZMA64547	skin	Vlieland Island	53.15N, 4.59E	KF946717
*Larus fuscus graelsii*	Tissue432	DNA sample	Leeuwarden/Ljouwert	53.13N, 5.45E	KF946718
*Larus fuscus intermedius*	Tissue327	DNA-sample	Leeuwarden/Ljouwert	53.13N, 5.45E	KF946719
*Larus fuscus intermedius*	ZMA55932	skin	Neeltje Jans	51.37N, 3.41E	KF946720
*Larus fuscus intermedius*	ZMA56230	skin	Europoort	51.56N, 4.05E	KF946721
*Larus fuscus intermedius*	ZMA58834	skin	Leeuwarden/Ljouwert	53.13N, 5.45E	KF946722
*Larus glaucoides glaucoides*	ZMA58836	wings	Texel Island	53.04N, 4.43E	KF946723
*Larus hyperboreus*	ZMA56221	skin	Texel Island	53.04N, 4.43E	KF946724
*Larus melanocephalus*	ZMA57226	skin	Wijdenes	52.37N, 5.1E	KF946725
*Larus michahellis michahellis*	ZMA58835	skin	Afsluitdijk	52.57N, 5.04E	KF946726
*Limosa lapponica lapponica*	ZMA58202	skin	Schiermonnikoog Island	53.29N, 6.11E	KF946727
*Limosa lapponica lapponica*	ZMA58203	skin	Schiermonnikoog Island	53.29N, 6.11E	KF946728
*Limosa lapponica taymyrensis*	ZMA58204	skin	Paesens	53.24N, 6.06E	KF946729
*Limosa lapponica taymyrensis*	ZMA58205	skin	Paesens	53.24N, 6.06E	KF946730
*Limosa lapponica taymyrensis*	ZMA58206	skin	Paesens	53.24N, 6.06E	KF946731
*Limosa lapponica taymyrensis*	ZMA58207	skin	Paesens	53.24N, 6.06E	KF946732
*Limosa lapponica taymyrensis*	ZMA58208	skin	Paesens	53.24N, 6.06E	KF946733
*Limosa lapponica taymyrensis*	ZMA58782	wings	Castricum	52.32N, 4.36E	KF946734
*Limosa lapponica taymyrensis*	ZMA58783	wings	Castricum	52.32N, 4.36E	KF946735
*Limosa limosa limosa*	Tissue457	DNA sample	Leeuwarden/Ljouwert	53.13N, 5.45E	KF946736
*Limosa limosa limosa*	ZMA57227	skin	Holysloot	52.24N, 5.01E	KF946737
*Limosa limosa limosa*	ZMA58229	skin	Waterland	52.07N, 4.19E	KF946738
*Limosa limosa limosa*	ZMA58230	skin	Edam	52.32N, 5.01E	KF946739
*Limosa limosa limosa*	ZMA58231	skin	Leeuwarden/Ljouwert	53.13N, 5.45E	KF946740
*Limosa limosa limosa*	ZMA58232	skin	Leeuwarden/Ljouwert	53.13N, 5.45E	KF946741
*Locustella luscinioides luscinioides*	ZMA64557	skin	Castricum	52.32N, 4.36E	KF946742
*Locustella naevia naevia*	ZMA56675	skin	Almere	52.22N, 5.13E	KF946743
*Locustella naevia naevia*	ZMA56678	skin	Almere	52.22N, 5.13E	KF946744
*Locustella naevia naevia*	ZMA57235	skin	Westenschouwen	51.41N, 3.42E	KF946745
*Locustella naevia naevia*	ZMA58812	skin	Castricum	52.32N, 4.36E	KF946746
*Locustella naevia naevia*	ZMA58936	skin	Hondsbossche Zeewering	52.44N, 4.38E	KF946747
*Locustella naevia naevia*	ZMA60132	skin	Kennemerduinen	52.42N, 4.58E	KF946748
*Locustella naevia naevia*	ZMA60133	skin	Kennemerduinen	52.42N, 4.58E	KF946749
*Locustella naevia naevia*	ZMA64556	skin	Castricum	52.32N, 4.36E	KF946750
*Loxia curvirostra curvirostra*	ZMA57246	skin	Eesveen	52.5N, 6.06E	KF946751
*Loxia curvirostra curvirostra*	ZMA57247	skin	Leersum	52.01N, 5.25E	KF946752
*Luscinia megarhynchos megarhynchos*	ZMA58798	skin	Amsterdam	52.21N, 4.53E	KF946753
*Lymnocryptes minimus*	ZMA55930	skin	Heerhugowaard	52.4N, 4.51E	KF946754
*Lymnocryptes minimus*	ZMA58293	skin	Uitgeest	52.31N, 4.42E	KF946755
*Milvus milvus milvus*	ZMA58307	wings	Grolloo	52.55N, 6.39E	KF946756
*Milvus milvus milvus*	ZMA58824	wings	Susteren	51.03N, 5.52E	KF946757
*Milvus milvus milvus*	ZMA58825	skin	Heurne	51.54N, 6.34E	KF946758
*Motacilla alba yarrellii*	ZMA58946	skin	Haastrecht	51.59N, 4.46E	KF946759
*Motacilla cinerea cinerea*	ZMA57241	skin	Westenschouwen	51.41N, 3.42E	KF946760
*Motacilla cinerea cinerea*	ZMA58266	skin	Westenschouwen	51.41N, 3.42E	KF946761
*Motacilla cinerea cinerea*	ZMA58267	skin	Westenschouwen	51.41N, 3.42E	KF946762
*Motacilla cinerea cinerea*	ZMA58945	skin	Westenschouwen	51.41N, 3.42E	KF946763
*Muscicapa striata striata*	ZMA57336	skin	Ilpendam	52.27N, 4.56E	KF946764
*Numenius arquata arquata*	Tissue431	DNA sample	Leeuwarden/Ljouwert	53.13N, 5.45E	KF946765
*Numenius arquata arquata*	ZMA58765	skin	Schiermonnikoog Island	53.29N, 6.11E	KF946766
*Numenius arquata arquata*	ZMA58829	skin	Heemskerk	52.3N, 4.36E	KF946767
*Oenanthe oenanthe leucorhoa*	ZMA58868	skin	Leeuwarden/Ljouwert	53.13N, 5.45E	KF946768
*Oenanthe oenanthe oenanthe*	ZMA58275	skin	Hondsbossche Zeewering	52.44N, 4.38E	KF946769
*Oenanthe oenanthe oenanthe*	ZMA58800	skin	Noordbergum/Noardburgum	53.13N, 6E	KF946770
*Oriolus oriolus oriolus*	ZMA58288	skin	Heteren	51.57N, 5.45E	KF946771
*Oriolus oriolus oriolus*	ZMA58305	wings	Zundert	51.28N, 4.38E	KF946772
*Pandion haliaetus haliaetus*	ZMA58823	wing	Vlieland Island	53.15N, 4.59E	KF946773
*Panurus biarmicus biarmicus*	ZMA57318	skin	Oostvaardersdijk	52.29N, 5.23E	KF946774
*Panurus biarmicus biarmicus*	ZMA58262	skin	Lelystad	52.29N, 5.24E	KF946775
*Panurus biarmicus biarmicus*	ZMA58263	skin	Lelystad	52.29N, 5.24E	KF946776
*Panurus biarmicus biarmicus*	ZMA58264	skin	Lelystad	52.29N, 5.24E	KF946777
*Panurus biarmicus biarmicus*	ZMA58265	skin	Lelystad	52.29N, 5.24E	KF946778
*Panurus biarmicus biarmicus*	ZMA58854	skin	Oostvaardersdijk	52.29N, 5.23E	KF946779
*Panurus biarmicus biarmicus*	ZMA58855	skin	Oostvaardersdijk	52.29N, 5.23E	KF946780
*Panurus biarmicus biarmicus*	ZMA58856	skin	Oostvaardersdijk	52.29N, 5.23E	KF946781
*Parus ater ater*	Tissue555	DNA sample	Castricum	52.32N, 4.36E	KF946782
*Parus ater ater*	ZMA56219	skin	Huizen	52.17N, 5.14E	KF946783
*Parus ater ater*	ZMA57242	skin	Arnhem	51.58N, 5.53E	KF946784
*Parus ater ater*	ZMA57243	skin	Amsterdam	52.21N, 4.53E	KF946785
*Parus ater ater*	ZMA58867	skin	Amsterdam	52.21N, 4.53E	KF946786
*Parus ater ater*	ZMA64562	skin	Castricum	52.32N, 4.36E	KF946787
*Parus caeruleus caeruleus*	Tissue438	DNA sample	Castricum	52.32N, 4.36E	KF946788
*Parus caeruleus caeruleus*	Tissue439	DNA sample	Castricum	52.32N, 4.36E	KF946789
*Parus caeruleus caeruleus*	Tissue440	DNA sample	Castricum	52.32N, 4.36E	KF946790
*Parus caeruleus caeruleus*	ZMA58944	wing	Leeuwarden/Ljouwert	53.13N, 5.45E	KF946791
*Parus cristatus mitratus*	ZMA56677	skin	Nijverdal	52.22N, 6.28E	KF946792
*Parus cristatus mitratus*	ZMA57245	skin	Hoog Buurlo	52.1N, 5.5E	KF946793
*Parus major major*	ZMA58796	skin	Leeuwarden/Ljouwert	53.13N, 5.45E	KF946794
*Parus major major*	ZMA58797	skin	Castricum	52.32N, 4.36E	KF946795
*Parus palustris palustris*	ZMA57244	skin	Castricum	52.32N, 4.36E	KF946796
*Parus palustris palustris*	ZMA64561	skin	Goor	52.14N, 6.34E	KF946797
*Passer domesticus domesticus*	ZMA58799	skin	Cadier en Keer	50.49N, 5.46E	KF946798
*Passer domesticus domesticus*	ZMA60138	skin	Lekkerkerk	51.53N, 4.41E	KF946799
*Passer montanus montanus*	ZMA58851	skin	Zuidhorn	53.14N, 6.23E	KF946800
*Passer montanus montanus*	ZMA58852	skin	Zuidhorn	53.14N, 6.23E	KF946801
*Passer montanus montanus*	ZMA58853	skin	Zuidhorn	53.14N, 6.23E	KF946802
*Passer montanus montanus*	ZMA58950	skin	Zuidhorn	53.14N, 6.23E	KF946803
*Perdix perdix perdix*	ZMA58738	skin	Texel Island	53.04N, 4.43E	KF946804
*Perdix perdix perdix*	ZMA58739	skin	Petten	52.46N, 4.38E	KF946805
*Pernis apivorus*	ZMA58827	wings	Vledder	52.53N, 6.13E	KF946806
*Phalacrocorax aristotelis aristotelis*	ZMA58224	skin	Wijk aan Zee	52.28N, 4.34E	KF946807
*Philomachus pugnax*	ZMA56680	skin	Graftermeer polder	52.33N, 4.48E	KF946808
*Philomachus pugnax*	ZMA58250	skin	Lelystad	52.29N, 5.24E	KF946809
*Phoenicopterus chilensis*	ZMA56683	skin	Ransdorp	52.23N, 4.59E	KF946810
*Phoenicurus phoenicurus phoenicurus*	ZMA55914	skin	Westenschouwen	51.41N, 3.42E	KF946811
*Phylloscopus collybita collybita*	ZMA55917	skin	Nijverdal	52.22N, 6.28E	KF946812
*Phylloscopus collybita collybita*	ZMA55918	wings	Leveroy	51.14N, 5.5E	KF946813
*Phylloscopus collybita collybita*	ZMA56217	skin	Hoogland	52.1N, 5.21E	KF946814
*Phylloscopus trochilus*	ZMA58284	skin	Lelystad	52.29N, 5.24E	KF946815
*Phylloscopus trochilus*	ZMA58710	skin	Almere	52.22N, 5.13E	KF946816
*Phylloscopus trochilus*	ZMA58713	skin	Egmond aan Zee	52.37N, 4.38E	KF946817
*Phylloscopus trochilus*	ZMA58714	skin	Lekkerkerk	51.53N, 4.41E	KF946818
*Phylloscopus trochilus*	ZMA58715	skin	Texel Island	53.04N, 4.43E	KF946819
*Phylloscopus trochilus*	ZMA58716	skin	Castricum	52.32N, 4.36E	KF946820
*Phylloscopus trochilus*	ZMA58861	skin	Castricum	52.32N, 4.36E	KF946821
*Phylloscopus trochilus*	ZMA58933	wings	Goor	52.14N, 6.34E	KF946822
*Phylloscopus trochilus*	ZMA58934	skin	Eemshaven	53.26N, 6.52E	KF946823
*Picus viridis viridis*	ZMA58718	skin	Breda	51.33N, 4.46E	KF946824
*Picus viridis viridis*	ZMA58719	skin	Haaksbergen	52.08N, 6.4E	KF946825
*Picus viridis viridis*	ZMA58720	skin	Alkmaar	52.38N, 4.44E	KF946826
*Picus viridis viridis*	ZMA58721	skin	Roggel	51.17N, 5.54E	KF946827
*Picus viridis viridis*	ZMA58722	skin	Bergen	52.4N, 4.41E	KF946828
*Plectrophenax nivalis insulae*	ZMA56672	skin	Castricum	52.32N, 4.36E	KF946829
*Pluvialis apricaria*	ZMA58213	skin	Winsum	53.09N, 5.38E	KF946830
*Pluvialis apricaria*	ZMA58214	skin	Winsum	53.09N, 5.38E	KF946831
*Pluvialis apricaria*	ZMA58215	skin	Dronrijp/Dronryp	53.11N, 5.4E	KF946832
*Pluvialis squatarola squatarola*	ZMA56224	skin	Schiermonnikoog Island	53.29N, 6.11E	KF946833
*Pluvialis squatarola squatarola*	ZMA56225	skin	Schiermonnikoog Island	53.29N, 6.11E	KF946834
*Puffinus gravis*	ZMA64542	skin	Sexbierum/Seisbierrum	53.14N, 5.28E	KF946835
*Pyrrhula pyrrhula europoea*	ZMA56673	skin	Castricum	52.32N, 4.36E	KF946836
*Pyrrhula pyrrhula europoea*	ZMA58793	skin	Castricum	52.32N, 4.36E	KF946837
*Pyrrhula pyrrhula europoea*	ZMA58794	skin	Castricum	52.32N, 4.36E	KF946838
*Pyrrhula pyrrhula europoea*	ZMA58795	skin	Castricum	52.32N, 4.36E	KF946839
*Pyrrhula pyrrhula europoea*	ZMA60137	wings	Kennemerduinen	52.42N, 4.58E	KF946840
*Rallus aquaticus aquaticus*	ZMA58763	skin	Lauwersmeer	53.22N, 6.14E	KF946841
*Recurvirostra avosetta*	ZMA58216	skin	Petten	52.46N, 4.38E	KF946842
*Regulus ignicapilla ignicapilla*	Tissue448	DNA sample	Castricum	52.32N, 4.36E	KF946843
*Regulus ignicapilla ignicapilla*	ZMA57360	skin	Zundert	51.28N, 4.38E	KF946844
*Regulus ignicapilla ignicapilla*	ZMA58807	skin	Castricum	52.32N, 4.36E	KF946845
*Regulus ignicapilla ignicapilla*	ZMA58808	skin	Castricum	52.32N, 4.36E	KF946846
*Regulus regulus regulus*	ZMA64560	skin	Castricum	52.32N, 4.36E	KF946847
*Riparia riparia riparia*	ZMA58871	skin	Zeewolde	52.21N, 5.34E	KF946848
*Saxicola rubetra*	ZMA60131	skin	Kennemerduinen	52.42N, 4.58E	KF946849
*Saxicola rubetra*	ZMA64555	skin	Castricum	52.32N, 4.36E	KF946850
*Somateria mollissima mollissima*	ZMA58912	skin	Lauwersoog	53.24N, 6.12E	KF946851
*Stercorarius longicaudus*	ZMA58779	wings	Afsluitdijk	52.57N, 5.04E	KF946852
*Stercorarius longicaudus*	ZMA64546	skin	Petten	52.46N, 4.38E	KF946853
*Stercorarius parasiticus*	ZMA56229	skin	Vlieland Island	53.15N, 4.59E	KF946854
*Stercorarius parasiticus*	ZMA56684	wings	Terschelling Island	53.26N, 5.29E	KF946855
*Stercorarius parasiticus*	ZMA58778	skin	Den Oever	52.56N, 5.02E	KF946856
*Stercorarius parasiticus*	ZMA58830	skin	Den Helder	52.55N, 4.46E	KF946857
*Stercorarius pomarinus*	Tissue211	DNA sample	Texel Island	53.04N, 4.43E	KF946858
*Stercorarius pomarinus*	ZMA55929	skin	Hondsbossche Zeewering	52.44N, 4.38E	KF946859
*Stercorarius skua skua*	ZMA64545	skin	Egmond aan Zee	52.37N, 4.38E	KF946860
*Sterna albifrons albifrons*	ZMA58832	skin	Schiermonnikoog Island	53.29N, 6.11E	KF946861
*Sterna hirundo hirundo*	ZMA58915	skin	Eemshaven	53.26N, 6.52E	KF946862
*Sterna paradisaea*	ZMA58831	skin	Amsterdam	52.21N, 4.53E	KF946863
*Streptopelia decaocto decaocto*	ZMA58923	wing	Hoogkerk	53.12N, 6.3E	KF946864
*Streptopelia turtur turtur*	ZMA58757	skin	Texel Island	53.04N, 4.43E	KF946865
*Sylvia atricapilla atricapilla*	Tissue441	DNA sample	Castricum	52.32N, 4.36E	KF946866
*Sylvia atricapilla atricapilla*	Tissue442	DNA sample	Castricum	52.32N, 4.36E	KF946867
*Sylvia atricapilla atricapilla*	ZMA58268	skin	Bloemendaal	52.24N, 4.33E	KF946868
*Sylvia atricapilla atricapilla*	ZMA58269	skin	Bloemendaal	52.24N, 4.33E	KF946869
*Sylvia atricapilla atricapilla*	ZMA58270	skin	Bloemendaal	52.24N, 4.33E	KF946870
*Sylvia atricapilla atricapilla*	ZMA58759	skin	Cadier en Keer	50.49N, 5.46E	KF946871
*Sylvia borin borin*	Tissue443	DNA sample	Castricum	52.32N, 4.36E	KF946872
*Sylvia borin borin*	ZMA58758	skin	Groningen	53.14N, 6.35E	KF946873
*Sylvia borin borin*	ZMA58761	skin	Almere	52.22N, 5.13E	KF946874
*Sylvia borin borin*	ZMA58762	skin	Purmerend	52.28N, 4.58E	KF946875
*Sylvia communis communis*	ZMA55924	wing	Asten	51.21N, 5.48E	KF946876
*Sylvia communis communis*	ZMA57335	skin	Almere	52.22N, 5.13E	KF946877
*Sylvia communis communis*	ZMA58280	skin	Breda	51.33N, 4.46E	KF946878
*Sylvia communis communis*	ZMA58939	skin	Castricum	52.32N, 4.36E	KF946879
*Sylvia communis communis*	ZMA58940	skin	Bloemendaal	52.24N, 4.33E	KF946880
*Sylvia curruca blythi*	ZMA58941	skin	Houten	52.01N, 5.1E	KF946881
*Sylvia curruca blythi*	ZMA57237	skin	Rotterdam	51.57N, 4.32E	KF946882
*Sylvia curruca curruca*	ZMA55905	skin	Westenschouwen	51.41N, 3.42E	KF946883
*Sylvia curruca curruca*	ZMA55906	skin	Amsterdam	52.21N, 4.53E	KF946884
*Sylvia curruca curruca*	ZMA57328	skin	Almere	52.22N, 5.13E	KF946885
*Sylvia curruca curruca*	ZMA57329	skin	Texel Island	53.04N, 4.43E	KF946886
*Sylvia curruca curruca*	ZMA58282	skin	Zeewolde	52.21N, 5.34E	KF946887
*Sylvia curruca curruca*	ZMA58806	skin	Leeuwarden/Ljouwert	53.13N, 5.45E	KF946888
*Sylvia curruca curruca*	ZMA58864	skin	Eemshaven	53.26N, 6.52E	KF946889
*Sylvia curruca curruca*	ZMA58942	skin	Bloemendaal	52.24N, 4.33E	KF946890
*Sylvia nisoria nisoria*	ZMA58273	skin	Westenschouwen	51.41N, 3.42E	KF946891
*Tringa ochropus*	ZMA64544	skin	Castricum	52.32N, 4.36E	KF946892
*Tringa totanus totanus*	ZMA58212	skin	Schiermonnikoog Island	53.29N, 6.11E	KF946893
*Troglodytes troglodytes troglodytes*	Tissue447	DNA sample	Castricum	52.32N, 4.36E	KF946894
*Troglodytes troglodytes troglodytes*	ZMA58281	skin	Bloemendaal	52.24N, 4.33E	KF946895
*Turdus iliacus iliacus*	ZMA58287	skin	Bloemendaal	52.24N, 4.33E	KF946896
*Turdus merula merula*	ZMA56669	skin	Haarlem	52.23N, 4.37E	KF946897
*Turdus merula merula*	ZMA56670	skin	Bergen	52.4N, 4.41E	KF946898
*Turdus merula merula*	ZMA57345	skin	Zwolle	52.3N, 6.06E	KF946899
*Turdus merula merula*	ZMA58731	skin	Alkmaar	52.38N, 4.44E	KF946900
*Turdus merula merula*	ZMA58732	skin	Maasbree	51.21N, 6.03E	KF946901
*Turdus merula merula*	ZMA58733	skin	Maasbree	51.21N, 6.03E	KF946902
*Turdus merula merula*	ZMA58734	skin	Steijl	51.2N, 6.07E	KF946903
*Turdus merula merula*	ZMA58736	skin	Schiermonnikoog Island	53.29N, 6.11E	KF946904
*Turdus philomelos philomelos*	Tissue453	DNA sample	Leeuwarden/Ljouwert	53.13N, 5.45E	KF946905
*Turdus philomelos philomelos*	Tissue454	DNA sample	Leeuwarden/Ljouwert	53.13N, 5.45E	KF946906
*Turdus torquatus torquatus*	ZMA56222	skin	Texel Island	53.04N, 4.43E	KF946907
*Turdus torquatus torquatus*	ZMA56671	skin	Castricum	52.32N, 4.36E	KF946908
*Turdus torquatus torquatus*	ZMA58693	skin	Apeldoorn	52.1N, 5.58E	KF946909
*Turdus torquatus torquatus*	ZMA58694	skin	Vlieland Island	53.15N, 4.59E	KF946910
*Turdus torquatus torquatus*	ZMA58695	skin	Zuilichem	51.48N, 5.07E	KF946911
*Turdus torquatus torquatus*	ZMA64554	skin	Texel Island	53.04N, 4.43E	KF946912
*Turdus viscivorus viscivorus*	ZMA60130	skin	Kennemerduinen	52.42N, 4.58E	KF946913
*Tyto alba alba*	ZMA56233	skin	Burgerbrug	52.45N, 4.42E	KF946914
*Tyto alba guttata*	ZMA56682	skin	Wierden	52.22N, 6.34E	KF946915
*Tyto alba guttata*	ZMA58235	skin	Texel Island	53.04N, 4.43E	KF946916
*Tyto alba guttata*	ZMA58236	skin	Ouderkerk aan de Amstel	52.17N, 4.56E	KF946917
*Tyto alba guttata*	ZMA58843	skin	Westzaan	52.26N, 4.46E	KF946918
*Tyto alba guttata*	ZMA58844	skin	Zaanstreek	52.28N, 4.44E	KF946919
*Tyto alba guttata*	ZMA58845	skin	Roodkerk/Readtsjerk	53.15N, 5.55E	KF946920
*Tyto alba guttata*	ZMA58846	skin	Garijp/Garyp	53.1N, 5.57E	KF946921
*Tyto alba guttata*	ZMA58847	skin	Middenmeer	52.48N, 4.59E	KF946922
*Tyto alba guttata*	ZMA58848	wings	Leeuwarden/Ljouwert	53.13N, 5.45E	KF946923
*Tyto alba guttata*	ZMA58919	skin	Texel Island	53.04N, 4.43E	KF946924
*Tyto alba guttata*	ZMA64550	skin	Purmerend	52.28N, 4.58E	KF946925
*Tyto alba guttata*	ZMA64551	skin	Goor	52.14N, 6.34E	KF946926
*Uria aalge albionis*	ZMA56227	skin	Amsterdam	52.21N, 4.53E	KF946927
*Uria aalge albionis*	ZMA58218	skin	Vlieland Island	53.15N, 4.59E	KF946928
*Uria aalge albionis*	ZMA58916	skin	Petten	52.46N, 4.38E	KF946929
*Vanellus vanellus*	ZMA58784	wing	Valkenburg	52.09N, 4.25E	KF946930
*Vanellus vanellus*	ZMA58785	wing	Valkenburg	52.09N, 4.25E	KF946931
*Vanellus vanellus*	ZMA58786	wing	Valkenburg	52.09N, 4.25E	KF946932
*Vanellus vanellus*	ZMA58787	wing	Valkenburg	52.09N, 4.25E	KF946933
*Vanellus vanellus*	ZMA58788	wing	Valkenburg	52.09N, 4.25E	KF946934
*Vanellus vanellus*	ZMA58789	wing	Valkenburg	52.09N, 4.25E	KF946935
*Vanellus vanellus*	ZMA58790	wing	Valkenburg	52.09N, 4.25E	KF946936
*Vanellus vanellus*	ZMA58791	wing	Valkenburg	52.09N, 4.25E	KF946937

**Supplementary table 2.** Bird species (gulls *Larus* and skuas *Stercorarius*) from the Netherlands with low (< 1.1%) K2P-mean intraspecific distances.

Collection number and species		Collection number and species		Distance (%)
#ZMA58835	*Larus michahellis*	#Tissue327	*Larus fuscus*	0
#ZMA58835	*Larus michahellis*	#Tissue432	*Larus fuscus*	0
#ZMA58835	*Larus michahellis*	#ZMA55932	*Larus fuscus*	0
#ZMA58835	*Larus michahellis*	#ZMA56230	*Larus fuscus*	0
#ZMA64547	*Larus cachinnans*	#Tissue327	*Larus fuscus*	0
#ZMA64547	*Larus cachinnans*	#Tissue432	*Larus fuscus*	0
#ZMA64547	*Larus cachinnans*	#ZMA55932	*Larus fuscus*	0
#ZMA64547	*Larus cachinnans*	#ZMA56230	*Larus fuscus*	0
#ZMA64547	*Larus cachinnans*	#ZMA58835	*Larus michahellis*	0
#ZMA58921	*Larus argentatus*	#ZMA55932	*Larus fuscus*	0.14
#ZMA58921	*Larus argentatus*	#ZMA58835	*Larus michahellis*	0.14
#ZMA58921	*Larus argentatus*	#Tissue432	*Larus fuscus*	0.15
#ZMA58921	*Larus argentatus*	#ZMA56230	*Larus fuscus*	0.15
#ZMA64547	*Larus cachinnans*	#ZMA58834	*Larus fuscus*	0.15
#ZMA64547	*Larus cachinnans*	#ZMA58921	*Larus argentatus*	0.15
#ZMA58921	*Larus argentatus*	#Tissue327	*Larus fuscus*	0.16
#ZMA55932	*Larus fuscus*	#Tissue433	*Larus argentatus*	0.29
#ZMA58835	*Larus michahellis*	#Tissue433	*Larus argentatus*	0.29
#Tissue433	*Larus argentatus*	#Tissue432	*Larus fuscus*	0.30
#ZMA56230	*Larus fusca*	#Tissue433	*Larus argentatus*	0.30
#ZMA64545	*Stercorarius skua*	#ZMA55929	*Stercorarius pomarinus*	0.30
#ZMA58836	*Larus glaucoides*	#Tissue432	*Larus fuscus*	0.31
#ZMA58836	*Larus glaucoides*	#ZMA55932	*Larus fuscus*	0.31
#ZMA58836	*Larus glaucoides*	#ZMA56230	*Larus fuscus*	0.31
#ZMA58836	*Larus glaucoides*	#ZMA58835	*Larus michahellis*	0.31
#ZMA64547	*Larus cachinnans*	#Tissue433	*Larus argentatus*	0.31
#ZMA64547	*Larus cachinnans*	#ZMA58836	*Larus glaucoides*	0.31
#Tissue433	*Larus argentatus*	#Tissue327	*Larus fuscus*	0.32
#ZMA58836	*Larus glaucoides*	#Tissue327	*Larus fuscus*	0.32
#ZMA64545	*Stercorarius skua*	#Tissue211	*Stercorarius pomarinus*	0.43
#ZMA58835	*Larus michahellis*	#ZMA58834	*Larus fuscus*	0.45
#ZMA58836	*Larus glaucoides*	#ZMA58834	*Larus fuscus*	0.46
#ZMA58921	*Larus argentatus*	#ZMA58836	*Larus glaucoides*	0.46
#ZMA56221	*Larus hyperboreus*	#ZMA55932	*Larus fuscus*	0.58
#ZMA58835	*Larus michahellis*	#ZMA56221	*Larus hyperboreus*	0.58
#ZMA56221	*Larus hyperboreus*	#Tissue432	*Larus fuscus*	0.60
#ZMA56230	*Larus fuscus*	#ZMA56221	*Larus hyperboreus*	0.60
#ZMA58921	*Larus argentatus*	#ZMA58834	*Larus fuscus*	0.60
#ZMA64547	*Larus cachinnans*	#ZMA56221	*Larus hyperboreus*	0.61
#ZMA58836	*Larus glaucoides*	#Tissue433	*Larus argentatus*	0.62
#ZMA56221	*Larus hyperboreus*	#Tissue327	*Larus fuscus*	0.64
#ZMA58921	*Larus argentatus*	#ZMA56221	*Larus hyperboreus*	0.73
#ZMA58834	*Larus fuscus*	#Tissue433	*Larus argentatus*	0.75
#ZMA56221	*Larus hyperboreus*	#Tissue433	*Larus argentatus*	0.87
#ZMA58836	*Larus glaucoides*	#ZMA56221	*Larus hyperboreus*	0.93
#ZMA58834	*Larus fuscus*	#ZMA56221	*Larus hyperboreus*	1.06
